# Nutrient adequacy and environmental foot-print of Mediterranean, pesco-, ovo-lacto-, and vegan menus: a modelling study

**DOI:** 10.3389/fnut.2025.1681512

**Published:** 2025-11-11

**Authors:** Ángela Alcalá-Santiago, Noelia M. Rodríguez-Martín, Eduardo Casas-Albertos, José María Gálvez-Navas, Adela Castelló-Pastor, Belén García-Villanova, Esther Molina-Montes

**Affiliations:** 1 Department of Nutrition and Food Science, Faculty of Pharmacy, University of Granada, Granada, Spain; 2 Institute of Nutrition and Food Technology (INYTA) 'José Mataix', Biomedical Research Centre, University of Granada, Granada, Spain; 3 Instituto de Investigación Biosanitaria ibs.GRANADA, Granada, Spain; 4 Group of Plant Protein, Department of Food and Health, Instituto de la Grasa-CSIC, Seville, Spain; 5 CIBER de Epidemiología y Salud Pública (CIBERESP), Madrid, Spain; 6 Andalusian School of Public Health, Granada, Spain; 7 Department of Chronic Diseases, National Centre for Epidemiology, Carlos III Institute of Health, Madrid, Spain

**Keywords:** vegetarian diet, plant-based diet, diet design, environmental foodprint, nutritional quality

## Abstract

**Background:**

Consumer food choices are changing towards a more plant-based diet (PBD) due to growing awareness of their less detrimental effects on health and the environment. However, PBDs—particularly vegan diets—may lead to nutritional deficiencies compared to non-PBDs. These differences may, in part, be influenced by the typically lower caloric content of PBDs.

**Objective:**

To compare the nutritional adequacy and environmental footprints of four 7-day menu models (~ 2,000 kcal/day) Mediterranean (omnivorous) diet, two vegetarian-like diets, and one vegan.

**Methods:**

Menus were designed based on the recommendations of the Spanish Society for Community Nutrition (SENC) and Vegetarian Union (UVE), substituting animal-based foods with plant-based alternatives. Nutritional intake was assessed using the Spanish BEDCA food composition table (Base de Datos Española de Composición de Alimentos) and adequacy was evaluated using Dietary Reference Intakes (DRIs) and recommended intake levels. Daily environmental footprints were estimated with Agribalyse. Differences between the four diet models were evaluated by one-way ANOVA or Kruskal-Wallis tests, followed by post-hoc analyses to identify pairwise differences in nutrient intake (significance level: 95%).

**Results:**

Daily macronutrient intake did not differ substantially across the diet groups. Furthermore, all four menus met protein needs and most micronutrient DRIs. Shortfalls were observed for vitamin D and iodine across all diets, and for vitamin B₁₂ in the vegan model; *ω*-3 PUFA were below the 250 mg/day target in all diets, while saturated fat remained < 8% of total energy intake. Mean daily environmental footprints decreased progressively from omnivorous to vegan diets: −46% CO₂, −6.6% deprived water and −33% land use among others.

**Conclusion:**

Well-planned PBDs can achieve comparable sustainability and nutritional adequacy to a healthy Mediterranean diet, although attention is required to ensure adequate intake of certain micronutrients.

## 1 Introduction

The global adoption of plant-based diets (PBDs) is rising across diverse populations, driven by ethical, religious, health-related, social and environmental concerns (1–4). Transitioning from current western diets to PBDs is estimated to reduce diet-related greenhouse-gas emissions to 54–87% and lower premature mortality from non-communicable diseases by 18–21% (2, 5). Consequently, PBD adoption directly supports the 2030 Sustainable Development Goals (SDG), specifically SDG 3 (Good Health and Well-being), SDG 12 (Responsible Consumption and Production) and SDG 13 (Climate Action) (6). In line with these benefits, the Food and Agriculture Organization (FAO) and the World Health Organization (WHO) recommend a diet rich in fruits, vegetables, legumes, cereals, and nuts, with moderate consumption of fish, eggs, poultry, and dairy products, and limited consumption of red meats and starchy vegetables (5). Within this context, a dietary transition toward PBDs is taking place globally, as evidenced by declining consumption of animal-source foods such as meat, fish, eggs, and dairy, and a notable shift in the type of animal proteins consumed, with lower red-meat consumption and higher poultry intake (1, 2).

PBDs include both vegetarian and non-vegetarian diets, as well as vegan diets, which are the strictest form of PBDs. Vegan diet excludes all animal-based foods and avoids products that are made from or tested on animals (7). The vegetarian patterns comprise ovo-(and/or) lacto-vegetarian diets (which allow eggs and/or dairy), pesco-vegetarian diets (which include fish and seafood, along with eggs and/or dairy), and non-vegetarian patterns, such as the Mediterranean diet, which also aligns with plant-based (PB) principles. Indeed, the Mediterranean diet includes a high proportion of plant foods with reduced meat intake, which places this dietary pattern within a PB healthy omnivorous diet and along the “vegetarian-like” spectrum (8).

The vegetarian dietary patterns have been linked to lower risk of numerous diseases such as cardiovascular diseases mortality (9) and incidence of cerebrovascular events (10–12), diabetes, cancer, osteoporosis (13, 14), chronic kidney disease (15), and inflammation related to fibromyalgia (16).

However, while PBDs are widely considered health-promoting, they require careful planning and sufficient variety to deliver all essential nutrients (5, 17). Research on dietary intake and nutritional status among PBD consumers, particularly among vegans, remains inconsistent, making it difficult to draw firm conclusions on this issue (5, 18). Current evidence suggests that vegetarian and vegan adults consume less protein, vitamin B_12_, vitamin D, *ω*-3 series fatty acids [eicosapentaenoic acid (EPA) + docosahexaenoic acid (DHA)], iron, zinc, selenium, iodine and calcium than omnivores (5, 8, 18). In addition, according to some studies, vitamin B_12_, calcium and iodine are among the most compromised nutrients in vegans (18–20). However, PBDs can enhance the intake of other nutrients naturally present in PB foods such as fibre, polyunsaturated fatty acids (PUFAs) including *α*-linolenic acid (ALA), vitamin E, folate and magnesium, which are often underconsumed in omnivorous diets (5).

Because most comparative studies do not adjust nutrient intakes for total energy, lower-calorie vegan menus often appear deficient simply because they provide less energy, not because their nutrient density is inferior. Some studies have argued that well-planned vegetarian diets are nutritionally adequate by targeting foods rich in critical micronutrients, and, when necessary, using fortification or supplements (8, 21), but this issue remains controversial due to the methodological quality of the studies and the above given reason. Therefore, it is important to compare isoenergetic menus that represent well-defined patterns (ovo-lacto-vegetarian, pesco-vegetarian, vegan and omnivorous diet) in order to assess true nutrient equivalence.

Thus, this study aimed to compare the nutritional and sustainable quality of four isocaloric PBDs: Mediterranean omnivorous, pesco-vegetarian, ovo-lacto-vegetarian, vegan.

## 2 Methods

Study type: comparative diet modelling study.

### 2.1 Diet design

Four 7-day, isoenergetic menus (2,000 kcal/day: average daily energy requirement for adults) were constructed: (i) Mediterranean omnivorous; (ii) pesco-vegetarian; (iii) ovo-lacto-vegetarian; and (iv) vegan.

#### 2.1.1 Design of the omnivorous menu

The reference menu was designed in concordance with the Spanish Society of Community Nutrition (SENC) for adults (Supplementary Table S1) (22, 23). This diet model resembles the Mediterranean diet and a healthy diet; thus, the proposed approach is applicable to other dietary guidelines across different countries or populations. The consumption frequency of each food group, along with recommended portions and serving sizes, was taken into account for the diet design.

To design a diet that supplies about 2,000 kcal for an average adult, we chose the median values of the recommended portion size ranges given by SENC (24); this approach aligns with the target energy intake. For milk, yogurt, and nuts, portion sizes were instead drawn from typical household measures and the servings most commonly consumed by the Spanish population according to a modern Mediterranean diet (25). The theoretical quantities for the menu preparation, in the order they appear in the Supplementary Table S1, were: 250 mL of milk, 125 g of yogurt, 50 g of cured cheese, 100 g of fresh cheese, 50 g of bread, 70 g of pasta/rice, 175 g of potatoes, 175 g of vegetables, 150 g of fruit, 10 mL of olive oil, 70 g of legumes, 30 g of nuts, 110 g of meat, 130 g of fish, and 60 g of eggs.

To adjust portion sizes, diet calibrations were conducted on the third day of the menu to check the diet design. i.e., the energy content was reviewed and modified, if needed, to remain within ± 10% of the target caloric intake (2,000 kcal). The omnivorous diet plan, which served as the basis for developing the vegetarian diets (pesco-vegetarian and ovo-lacto-vegetarian) and vegan diet is shown in Table 1.

**Table 1:** Detailed 7-day standard omnivorous menu plan with dish names, ingredients, and portion quantities by meal.

Eating occasion	Monday	Tuesday	Wednesday	Thursday	Friday	Saturday	Sunday
Breakfast	Coffee with **milk (100 mL),** whole wheat toast (60 g) with tomato (50 g) and EVOO (5 g) and kiwi (150 g)	**Milk (150 mL)** with oats (30 g), kiwi (75 g), strawberries (75 g), and banana (75 g)	Coffee with **milk (100 mL)**, whole wheat toast (60 g) with tomato (50 g) and EVOO (5 g) and kiwi (150 g)	**Milk (150 mL)** with oats (30 g), kiwi (75 g), strawberries (75 g), banana (75 g), and nuts cream butter (30 g)	Coffee **with milk (100 mL)**, whole wheat toast (60 g) with tomato (50 g) and EVOO (5 g) and kiwi (150 g)	Coffee with **milk (100 mL)**, whole wheat toast (60 g) with tomato (50 g) and EVOO (5 g)	**Glass of milk (250 mL)**, whole wheat toast (60 g) with tomato (50 g) and EVOO (5 g) and kiwi (150 g)
Mid-morning	**Natural yogurt (125 g)** with oats (30 g) and banana (150 g)	Coffee with **milk (100 mL)**, whole wheat toast (60 g) with tomato (50 g) and EVOO (5 g), banana (75 g)	**Milk (150 mL)** with oats (30 g) and blueberries (150 g)	Coffee **with milk (100 mL)**, whole wheat toast (60 g) with tomato (50 g) and EVOO (5 g) and banana (75 g)	**Natural yogurt (125 g)** with oats (30 g), strawberries (75 g), and banana (75 g)	**Milk (150 mL)** with oats (30 g) with natural nuts cream butter (30 g) and blueberries (150 g)	**Natural yogurt (125 g)** with strawberries (150 g) and nuts (30 g)
Lunch	*Bean stew with vegetables* (75 g tomato, 75 g green pepper, 50 g onion, 50 g carrot, 150 g chard, 60 g beans, 150 g potato, 10 g EVOO) Bread (40 g) and apple (150 g)	*Pasta salad with vegetables and tomatoes sauce, tuna and cheese* (75 g eggplant, 75 g zucchini, 150 g tomato, 50 g onion, 60 g raw pasta, 10 g EVOO, **30 g fresh cheese**, 80 g canned tuna) Bread (40 g) and pear (150 g)	*Beef stew* (75 g tomato, 75 g green pepper, 50 g onion, 50 g carrot, 100 g potato, 150 g mushroom, **110 g beef**, 10 g EVOO) Bread (40 g) and mango (150 g)	*Eggs on a plate* **(60 g egg**, 100 g eggplant, 100 g zucchini, 50 g onion, 5 g garlic, 100 g tomato, 50 g peas, 10 g EVOO, **30 g cured ham)** Bread (40 g) and apple (150 g)	*Lentil stew with pumpkin and rice* (60 g lentils, 50 g onion, 75 g green pepper, 75 g tomato, 50 g zucchini, 50 g carrot, 150 g pumpkin, 60 g raw rice, 10 g EVOO) Bread (60 g) and pear (150 g)	*Grilled salmon* with sautéed quinoa and artichokes (**salmon 130 g**, 5 g garlic, 50 g onion, 150 g artichokes, 80 g quinoa, 10 g EVOO) Bread (60 g) and mango (150 g)	*Baked sea bream with vegetables and potatoes* (**130 g sea bream**, 50 g onion, 75 g green pepper, 75 g red pepper, 150 g potato, 75 g broccoli, 10 g EVOO) Bread (60 g) and apple (150 g)
Snack	Milk shaker with **milk (150 mL)** with natural nuts cream butter (30 g) and blueberries (150 g)	**Natural yogurt (125 g)** with kiwi (75 g), strawberries (75 g) and nuts (30 g)	**Natural yogurt (125 g)** with strawberries (150 g) and nuts (30 g)	**Natural yogurt (125 g)** with kiwi (75 g) and strawberries (75 g)	Milk shaker with **milk (150 mL)**, strawberries (75 g), banana (75 g) and nuts (30 g)	**Natural yogurt (125 g)** with kiwi (150 g) and banana (150 g)	Whole wheat toast (60 g) with tomato (50 g) and EVOO (5 g) and banana (150 g)
Dinner	*Grilled turkey fillet with sautéed rice and vegetables* (**110 g turkey**, 60 g raw rice, 75 g asparagus, 150 g mushrooms, 5 g garlic, 10 g EVOO, 75 g zucchini) Bread (40 g) and mandarin (150 g)	*Carrot puree* (150 g carrot, 50 g potato, 5 g EVOO) *Potato omelette* (**60 g egg,** 100 g potato, 5 g EVOO) Bread (40 g) and orange (150 g)	*Hummus toast* (60 g bread, 60 g chickpeas, 5 g EVOO) *Gratin cauliflower* (150 g cauliflower, **30 g grated cheese**) Grapes (150 g)	Grilled hake with sautéed green beans and potatoes (**Hake 130 g**, 200 g green beans, 100 g potatoes, 10 g EVOO) Bread (40 g) and mandarin (150 g)	*Chicken fajitas with mixed vegetables* (60 g corn tortillas, **110 g chicken**, 50 g onion, 75 g red pepper, 75 g green pepper, 10 g EVOO) Orange (150 g)	*Spinach and cheese tortilla sandwich* (60 g bread, **60 g egg**, 200 g spinach, 10 g EVOO, **30 g grated cheese**) Grapes (150 g)	*Minced meat soup* (**250 mL chicken broth,** 60 g egg, 40 g ham, 50 g toasted bread) *Salad* (75 g spinach, 50 g escarole, 75 g tomato, **40 g fresh cheese**, 25 g corn, 50 g grated carrot, 5 g EVOO) Mandarin (150 g)

Foods of animal origin (and the portion sizes) that were replaced throughout the 3 types of PBD are indicated bold. The names of the dishes are written in italics. EVOO: extra-virgin olive EVOO.

#### 2.1.2 Design of the plant-based menu

In the 7-day pesco-vegetarian diet menu, meat and its derivatives were substituted with PB foods, e.g., tofu and textured soy protein, or animal-based foods allowed in this diet, such as fish, eggs and cheese, accounting for nutritional quality intake.

In the vegan diet menu, all animal-based foods (meat, fish, eggs and dairy) were excluded. These were replaced exclusively by PB foods, such as PB meat alternatives (tofu, textured soy protein, seitan, tempeh), PB beverages, soy yogurt, seeds, and legumes or legume flours.

In all cases the SENC recommendations were followed for overall diet planning, while Spanish Vegetarian Union (UVE) guidelines were used for PB protein sources not included in the SENC recommendations (26).

Energy calibration was also performed for each day to ensure caloric adequacy. For each isocaloric menu, if the total energy deviated by more than ± 10% from the 2,000-kcal target, portion sizes were adjusted, or foods were modified, according to UVE recommendations for plant-protein foods to maintain energy value within the acceptable range.

Table 2 summarises every food change applied to generate the alternative menus. For each animal-derived item removed from the reference diet, the table lists the replacement food, PB or an allowable animal product, and its portion size. Substitutions are shown by day of the week (Monday–Sunday) and by eating occasion (e.g., breakfast, mid-morning snack, lunch).

**Table 2:** Substitutions of animal-based foods by PB foods applied to Table 1.

Menu rotation	Omnivorous	Pesco-vegetarian	Ovo-lacto-vegetarian	Vegan
Daily	Milk (250 mL) and yogurt (125 g)	-	-	Soy drink (250 mL) and soy yogurt (125 g)
Monday	Turkey fillet (100 g)	Tofu (100 g)	Tofu (100 g)	Tofu (100 g)
Tuesday	Canned tuna (80 g)	-	Textured soy (40 g) and grated cheese (30 g)	Textured soy (40 g)
	Egg (60 g)	-	-	Chickpea flour (40 g)
Wednesday	Beef (100 g)	Cod (125 g)	Seitan (100 g)	Seitan (100 g)
	Grated cheese, mozzarella (30 g)	-	-	Soybean sprouts (50 g) and flax seeds (10 g)
Thursday	Egg (60 g) and ham (30 g)	Grated cheese, mozzarella (30 g)	Grated cheese, mozzarella (30 g)	Peas (60 g)
	Hake (125 g)	-	Seitan (85 g) and chickpeas (30 g)	Seitan (85 g) and chickpeas (30 g)
Friday	Chicken (100 g)	Textured soy (40 g) and grated cheese, mozzarella (30 g)	Textured soy (40 g) and grated cheese, mozzarella (30 g)	Textured soy (40 g)
Saturday	Salmon (125 g)	-	Tofu (100 g) and flax seeds (10 g)	Tofu (100 g) and flax seeds (10 g)
	Egg (60 g)	-	-	Chickpea flour (40 g)
Sunday	Sea bream (125 g)	-	Tempeh (100 g)	Tempeh (100 g)
	Chicken broth (250 mL), egg (60 g), ham (30 g) and fresh cheese (40 g)	Vegetable broth (250 mL), chickpeas (30 g) and flax seeds (10 g)	Vegetable broth (250 mL), chickpeas (30 g) and flax seeds (10 g)	Vegetable broth (250 mL), soy sprouts (50 g), lentils (30 g) and flax seeds (10 g)

### 2.2 Assessment of compliance with dietary guidelines

The alignment of the 7-day menus was evaluated by analysing serving sizes and intake frequencies in comparison with the reference dietary guidelines (SENC 2018 and UVE), as detailed in Supplementary Table S2. More specifically, we considered food groups with their respective intake frequencies (daily or weekly) based on the aforementioned guidelines. The food groups considered from the SENC Dietary Guidelines were: milk and dairy products; bread, cereals, rice, pasta, and potatoes; vegetables; fruits; olive oil; legumes; nuts; fish and seafood; lean meats; and eggs. The food groups included in the UVE recommendations were: legumes, soy and its derivatives, seitan, and seeds. Although legumes appear as a separate group in the SENC Dietary Guidelines, the UVE framework classifies them (along with soy foods and seitan) under a single “legumes and derivatives” category. To maintain internal consistency, we applied the UVE criteria for this food group when designing the PB menus. Thus, for legumes, the intake frequency from the SENC guidelines was applied to the omnivorous diet, while for the PBDs, legume intake frequency was evaluated together with soy-based foods and seitan.

The four menus met all the recommended consumption frequencies from the SENC Dietary Guidelines (except for animal-based foods not included in the PBDs) and the UVE recommendations, supporting that all were healthy and balanced.

### 2.3 Assessment of energy and nutrient intake

To compare the macro- and micronutrient intake of the four diet types, two different Food Composition Tables (FCTs) were used: All foods present in the Spanish BEDCA database (Base de Datos Española de Composición de Alimentos) were transferred to an Excel spreadsheet (27). Foods not included in BEDCA, such as tempeh, seitan, and soy yogurt, were added using USDA (United States Department of Agriculture) nutritional data (28). Once the spreadsheet was complete with all foods and their respective nutritional information, the daily diets were added in columns (7 columns for each diet, totalling 7×4 = 28 columns). Formulas were applied to each nutrient column to calculate the energy and nutrient contribution for each food and day (food in g x nutrient/100 g of food). While foods were considered as whole items (including both edible and inedible parts) in diet planning, only the edible part was considered when compiling nutrient values, as indicated in the FCT. For instance, for 150 g kiwi, the edible portion considered was 132 g (edible portion: 0.82).

To distinguish between nutrients derived from PB and animal-based food sources, we estimated the percentage contribution of nutrients by dietary source. As for iron, we assumed that 40% of the total iron in meat, poultry, and fish was heme iron, with the remaining 60% classified as non-heme iron. The same was considered for eggs and dairy products. Iron from PB sources was considered entirely non-heme (29).

### 2.4 Diet quality and adequacy assessment

To assess diet quality, a comparison based on Nutritional Goals (NGs) established by the SENC, the FAO, and the European Food Safety Authority (EFSA) were used, specifically regarding the lipid profile of the diet and other key targets (Supplementary Table 3) (30–32). In addition, Nutrient Reference Values (NRV) (33) were considered to assess nutrient intake for an adult with an average energy requirement of 2,000 kcal.

Other nutrient intake recommendations, namely the Dietary Reference Intakes (DRIs) from the Institute of Medicine (IOM) (34), for age and gender-specific recommendations were applied (35–41). Only the adult categories of 19–30, 31–50, and 51–70 years, for both women and men, were considered. Nutritional adequacy was assessed by calculating the percentage of the Recommended Dietary Allowance (RDA) covered by the average daily intake (of the 7-day menu) for each nutrient. Nutrient intakes meeting the RDA levels (>100% compliance) were considered adequate; otherwise, the intake of nutrient was considered potentially inadequate. Tolerable Upper intake levels (ULs) were also considered to account for potential over-adequacy. In addition, macronutrient intake was evaluated based on the Acceptable Macronutrient Distribution Ranges (AMDRs) established by IOM, which states that carbohydrates provide 45–65% of total energy, fats 20–35%, and proteins the remaining part of total energy (10–35%).

### 2.5 Methodology for compiling footprints indicators

A cradle-to-home life-cycle boundary was adopted. Included processes comprised primary production (crop, livestock, aquaculture or wild fishery). The edible portion weights of single ingredients in the menu were considered.

Environmental-intensity factors were obtained from Agribalyse 3.1.1 (42), in the case of missing foods (e.g., mushrooms), values from Robinson *et al*. 2019 (43) were used. The final daily footprints were calculated by summing the mass of each edible food ingredient (g) multiplied by its corresponding intensity factor. Water and land footprints were computed analogously. Other footprint indicators reported in AGRIBALYSE 3.1.1 were also included in this study (climate change, ozone depletion, ionizing radiation, photochemical ozone formation, fine particulate matter, human toxicity—non-carcinogenic substances, human toxicity—carcinogenic substances, terrestrial and freshwater acidification, freshwater eutrophication, marine eutrophication, terrestrial eutrophication, freshwater ecotoxicity, land use, water resource depletion, energy resource depletion, mineral resource depletion).

### 2.6 Statistical data analysis

For each food and nutrient, the seven daily values per menu were treated as independent replicates (*n* = 7). Intakes were expressed as mean ± SD. Normality was checked with the Shapiro–Wilk test and homogeneity of variances with Levene’s test. If both assumptions held, a one-way ANOVA (factor = dietary pattern, 4 levels) was performed; otherwise, a Kruskal–Wallis test replaced ANOVA. When the overall test was significant (*p* < 0.05), pairwise differences were explored with Tukey’s HSD (parametric), or Dunn’s test (non-parametric) with Benjamini-Hochberg (BH) multiple testing correction. Analyses were run in Python 3.11 with SciPy 1.11, statsmodels 0.14 and scikit-posthocs 0.7, and in R version 4.4 (44).

## 3 Results

### 3.1 Nutrient distribution in isoenergetic menus

Table 3 shows the nutritional profile of the menus. All 7-day menus were isocaloric, delivering 2,000 kcal/day, and all complied with the macronutrient ranges proposed by the Spanish NGs and EFSA for a balanced diet (10–15% of energy from protein, <40% from fat, and >50% from carbohydrates). The macronutrient distribution also met those set as AMDR by the IOM. No statistically significant differences were seen among diets for total fat, protein or carbohydrate energy contribution. Interestingly, the vegan menu supplied a significantly higher amount of dietary fibre (>10 g/day) than the other three patterns (*p* = 0.02). All four menus provided fibre intakes above the NG recommendations (>14 g/d per 1,000 kcal).

**Table 3:** Macronutrients (mean ± SD; *n* = 7 days) presented in the isocaloric menus for omnivorous, pesco-vegetarian, ovo-lacto-vegetarian and vegan diets.

Nutrient	Omnivorous (mean ± SD)	Pesco-vegetarian (mean ± SD)	Ovo-lacto-vegetarian (mean ± SD)	Vegan (mean ± SD)	*p*-value^1^
Energy (kcal)	2146.47 ± 186.35	2078.36 ± 193.82	2113.77 ± 173.60	2197.59 ± 201.07	0.684
Fat (g)	92.68 ± 32.41	80.08 ± 8.46	80.80 ± 8.56	72.97 ± 7.43	0.166
Protein (g)	91.97 ± 11.91	88.16 ± 11.85	88.64 ± 10.32	91.31 ± 15.06	0.952
Carbs (g)	263.13 ± 35.95	251.33 ± 33.97	256.41 ± 34.57	293.58 ± 24.42	0.096
Fiber (g)	45.76 ± 9.36^b^	45.18 ± 5.72^b^	46.54 ± 5.56^b^	56.35 ± 6.80^a^	0.020
Fat (% of E)	39.26 ± 15.07^b^	34.78 ± 3.27^b^	34.50 ± 3.64^b^	29.87 ± 0.78^a^	0.036
Protein (% of E)	17.15 ± 1.75	16.96 ± 1.53	16.78 ± 1.50	16.58 ± 1.82	0.757
Carbs (% of E)	48.93 ± 3.99^b^	48.27 ± 3.49^b^	48.43 ± 4.20^b^	53.51 ± 2.28^a^	0.056

^1^ *p*-values derived from one-way ANOVA or Kruskal-Wallis, where appropriate. Pairwise comparisons were performed via Tukey (parametric) or Dunn (non-parametric) post-hoc tests, with *p*-values corrected for multiple testing through BH. Means sharing a common superscript letter do not differ significantly between the groups (*p* < 0.05).

The macronutrient profile of all four menus was relatively similar. The only notable difference was the vegan menu, which supplied less than 30% of total energy as fats, whereas it ranged from 34–39% in the other diet groups. Moreover, this difference was statistically significant. No significant differences were observed for protein or carbs relative to energy intake between the diet groups.

With respect to the vitamin and mineral profiles of the isoenergetic menus (Table 4), statistically significant differences emerged for iodine (*p* = 0.003), selenium (*p* = 0.002), vitamin B_3_ (*p* < 0.001), vitamin B_12_ (*p* = 0.007), vitamin D (*p* = 0.001) and sodium (*p* = 0.042); in all cases the vegan menu provided the lowest intake compared to the others diet groups (*p* < 0.05). In contrast, the same vegan pattern provided higher amounts of several micronutrients, reaching statistical significance for vitamin B_1_ (*p* = 0.014) and iron (*p* = 0.026) and showing non-significant upward trends for folate (*p* = 0.257), potassium (*p* = 0.401) and magnesium (*p* = 0.164).

**Table 4:** Micronutrients: vitamins and minerals (mean ± SD; *n* = 7 days) presented in the isocaloric menus for omnivorous, pesco-vegetarian, ovo-lacto-vegetarian and vegan diets.

Nutrient	Omnivorous (mean ± SD)	Pesco-vegetarian (mean ± SD)	Ovo-lacto-vegetarian (mean ± SD)	Vegan (mean ± SD)	*p*-value^1^
Vit B₁ (mg)	1.90 ± 0.16^b^	1.78 ± 0.18^b^	1.79 ± 0.17^b^	2.16 ± 0.17^a^	0.001
Vit B₂ (mg)	2.19 ± 0.32	2.10 ± 0.20	2.13 ± 0.20	2.40 ± 0.25	0.119
Vit B₃ (mg NE)	28.74 ± 4.12^a^	26.62 ± 2.82^a,b^	22.80 ± 1.22^b-c^	21.62 ± 2.16^c^	<0.001
Vit B₅ (mg)	1.17 ± 0.76	1.16 ± 0.63	1.14 ± 0.56	1.50 ± 0.41	0.634
Vit B₆ (mg)	3.30 ± 0.35	3.21 ± 0.26	3.09 ± 0.35	3.16 ± 0.34	0.667
Vit B₉ (μg)	590.66 ± 127.86	594.53 ± 90.42	606.83 ± 64.55	716.59 ± 123.15	0.103
Vit B₁₂ (μg)	3.65 ± 1.84^a^	4.31 ± 2.24^a^	2.90 ± 1.11^a^	0.30 ± 0.63^b^	<0.001
Vit C (mg)	435.25 ± 112.09	436.16 ± 110.77	436.53 ± 110.57	432.43 ± 114.08	0.985
Vit D (μg)	2.85 ± 4.88^a^	3.82 ± 5.06^a^	0.86 ± 0.50^a^	0.00 ± 0.00^b^	0.001
Vit E (mg *α*-TE)	16.81 ± 3.08	17.00 ± 2.84	16.72 ± 2.09	18.56 ± 2.39	0.253
Retinol (μg)*	1436.73 ± 634.78	1470.01 ± 614.38	1462.64 ± 605.29	1334.58 ± 588.12	0.974
Potassium (mg)	5265.68 ± 668.85	5246.44 ± 562.88	5232.86 ± 586.16	5707.41 ± 586.21	0.401
Calcium (mg)	1158.09 ± 240.47	1258.96 ± 113.81	1312.88 ± 175.90	1190.82 ± 235.09	0.146
Sodium (mg)	1820.25 ± 495.65^c^	1643.30 ± 238.54^c^	1581.46 ± 242.48^b-c^	1270.61 ± 243.13^a,b^	0.042
Iron (mg)	19.57 ± 3.77^c^	19.77 ± 4.33^b-c^	20.83 ± 3.85^a,b^	26.12 ± 4.92^a^	0.026
Iodine (μg)	119.52 ± 16.68^a^	143.61 ± 61.52^a^	109.20 ± 18.67^a,b^	55.16 ± 20.87^b^	0.003
Selenium (μg)	105.54 ± 22.53^a^	101.86 ± 11.30^a,b^	83.79 ± 8.47^b-c^	76.52 ± 11.51^c^	0.002
Magnesium (mg)	544.42 ± 174.04	533.47 ± 102.20	555.36 ± 119.97	680.88 ± 153.71	0.164
Phosphorus (mg)	1703.54 ± 145.60	1752.27 ± 169.48	1741.37 ± 182.36	1601.28 ± 246.49	0.511
Zinc (mg)	11.52 ± 1.58	10.93 ± 1.48	11.11 ± 1.48	10.96 ± 1.90	0.899

^1^ *p*-values derived from one-way ANOVA or Kruskal-Wallis, where appropriate. Pairwise comparisons were performed via Tukey (parametric) or Dunn (non-parametric) post-hoc tests, with *p*-values corrected for multiple testing through BH. Means sharing a common superscript letter do not differ significantly between the groups (*p* < 0.05). ^*^ Retinol (preformed Vitamin A).

When the fatty-acid profiles of the four diet groups (7-day menus) were compared, as shown in Table 5, significant differences were apparent for total saturated fatty acids (SFA, *p* = 0.001); the vegan menu provided markedly less SFA (9.36 ± 1.13 g/day) than the other patterns (18.1–19.9 g/day). A non-significant increasing trend was observed for PUFA in the vegan diet (*p* = 0.082), whereas monounsaturated fatty-acid (MUFA) intake did not differ across the menus. At the individual-fatty-acid level, intake of stearic, lauric and myristic acids was significantly lower in the vegan menu, whereas intakes of linoleic acid (LA, 18:2 *ω*-6) and alpha-linolenic (ALA, 18:3 ω-3) were significantly higher in this group than in the other three diet patterns. Of note, intake of EPA was similar among pesco-vegetarians (0.12 ± 0.17 g/day) and the omnivorous diet group (0.13 ± 0.17 g/day). The intake of DHA was similar among the pesco-vegetarian diet.

**Table 5:** Fatty acid profile and cholesterol composition in the isocaloric menus for omnivorous, pesco-vegetarian, ovo-lacto-vegetarian and vegan diets.

Nutrient	Omnivorous (mean ± SD)	Pesco-vegetarian (mean ± SD)	Ovo-lacto-vegetarian (mean ± SD)	Vegan (mean ± SD)	*p*-value^1^
SFA (g)	19.90 ± 4.54^a^	19.24 ± 2.87^a^	18.96 ± 2.49^a^	9.36 ± 1.13^b^	<0.001
MUFA (g)	32.11 ± 2.73	32.53 ± 3.56	32.42 ± 3.07	29.00 ± 2.58	0.114
PUFA (g)	22.34 ± 4.25	22.47 ± 2.17	22.95 ± 2.55	25.31 ± 3.11	0.082
LA, 18:2 n-6 (g)	13.65 ± 0.87^b^	13.98 ± 1.42^a,b^	14.83 ± 1.96^a,b^	17.07 ± 2.26^a^	0.034
ALA, 18:3 n-3 (g)	0.73 ± 0.10^b^	0.81 ± 0.28^a,b^	0.93 ± 0.36^a,b^	1.20 ± 0.36^a^	0.019
AA, 20:4 n-6 (g)	0.03 ± 0.03	0.01 ± 0.02	0.00 ± 0.00	0.00 ± 0.00	0.062
EPA, 20:5 n-3 (g)	0.13 ± 0.17^a^	0.13 ± 0.17^a^	0.01 ± 0.00^a,b^	0.01 ± 0.00^b^	0.006
DHA, 22:6 n-3 (g)	0.23 ± 0.31	0.23 ± 0.31	0.01 ± 0.00	0.01 ± 0.00	0.189
Lauric acid (12:0) (g)	0.21 ± 0.08^a^	0.21 ± 0.00^a^	0.21 ± 0.00^a^	0.00 ± 0.00^b^	0.001
Myristic acid (14:0) (g)	0.72 ± 0.28^a^	0.71 ± 0.09^a^	0.66 ± 0.00^a,b^	0.02 ± 0.00^b^	0.001
Stearic acid (18:0) (g)	2.12 ± 0.21^a^	2.02 ± 0.14^a^	1.99 ± 0.19^a,b^	1.53 ± 0.19^b^	0.001
Cholesterol (mg) ^¥^	227.90 ± 105.36^a^	211.00 ± 122.87^a^	174.43 ± 107.13^a,b^	0.00 ± 0.00^b^	0.001
LA: ALA^2, ¥^	18.69	17.26	15.94	14.23	
PUFA: SFA^2, ¥^	1.12	1.17	1.21	2.70	
(MUFA+PUFA): SFA^2, ¥^	2.73	2.86	2.92	5.80	

^1^ *P*-values derived from one-way ANOVA or Kruskal-Wallis, where appropriate. Pairwise comparisons were performed via Tukey (parametric) or Dunn (non-parametric) post-hoc tests, with *p*-values corrected for multiple testing through BH. Means sharing a common superscript letter do not differ significantly between the groups (*p* < 0.05). ^2^ Calculated ratios. ¥ Diet quality indicators based on NGs established by SENC (Supplementary Table 2): cholesterol: < 300 mg/d; LA: ALA: 4/1–5/1; PUFA: SFA: ≥ 0.5; (MUFA+PUFA): SFA: ≥ 2. SFA: saturated fatty acids; MUFA: monounsaturated fatty acids; PUFA: polyunsatured fatty acids; LA: linoleic acid: ALA: linolenic acid; AA: araquidonic acid; EPA: eicosapentanoic acid; DHA: docosahexaenoic acid.

### 3.2 Diet quality indices of the menus

Supplementary Tables S3, S5 show the comparison of the four types of diet in relation to compliance with the NGs for the Spanish population. It was observed that all met most of the quality indices set in these guidelines. Compliance with the intake of fiber and of fruits and vegetables, was similar across the four diets and above the reference value. The contribution of PUFAs to total energy intake was above the 5% threshold across all four diet groups. The intake of SFAs met the objectives, with the vegan diet showing the greatest compliance. The percentage of *ω*-3 and ω-6 fatty acids in the diet relative to the total energy intake did not vary notably among the diet groups; none met the recommendations for ω-3 fatty acids. As a consequence, the ω-6/ω-3 ratio was markedly high in all diet groups. Finally, all complied with the threshold given for cholesterol; as expected, the amount of cholesterol was negligible in the vegan diet. As for the Ca/P ratio, this index showed a higher trend for the intake of *P* in all diet groups. As also shown in Table 4, intakes over 1,000 mg/day of calcium and 400 μg/day of folate were met in the four groups.

### 3.3 Nutrient adequacy in adult men and women

Nutritional adequacy by sex for the age group 19 to 30 years is shown in Table 6. Those given for age groups 31 to 50, and 51 to 70, are shown in Supplementary Tables S4, S5, respectively.

**Table 6:** Nutritional adequacy in adult (19–30 years) men and women (% of RDA) presented in the 7-day isocaloric menu (2,000 kcals) for each diet.

	Men					Women				
Nutrient	Omnivorous	Pesco-Vegetarian	Ovo-lacto-Vegetarian	Vegan	RDA	Omnivore	Pesco-Vegetarian	Ovo-lacto-Vegetarian	Vegan	RDA
Energy (kcal)	107	104	106	110	2000	107	104	106	110	2000
Protein (g)	164	157	158	163	56	200	192	193	199	46
Total carbohydrate (g)	202	193	197	226	130	202	193	197	226	130
Dietary fibre (g)	120	119	123	148	38	183	181	186	225	25
Vit B₁ (mg)	158	148	149	180	1.2	173	162	163	196	1.1
Vit B₂ (mg)	168	162	164	185	1.3	199	191	194	218	1.1
Vit B₃ (mg NE)	180	166	143	135	16	205	190	163	154	14
Vit B₅ (mg) ¥	23	23	23	30	5	23	23	23	30	5
Vit B₆ (mg)	254	247	238	243	1.3	254	247	238	243	1.3
Vit B₉ (μg)	148	149	152	179	400	148	149	152	179	400
Vit B₁₂ (μg)	152	180	121	13	2.4	152	180	121	13	2.4
Vit C (mg)	484	485	485	480	90	580	582	582	577	75
Vit D (μg)	19	25	6	0	15	19	25	6	0	15
Vit E (mg α-TE)	112	113	111	124	15	112	113	111	124	15
Vit A (μg)*	160	163	163	148	900	205	210	209	191	700
Potassium (mg) ¥	155	154	154	168	3,400	203	202	201	220	2,600
Calcium (mg)	116	126	131	119	1,000	116	126	131	119	1,000
Sodium (mg) ¥	118	110	105	85	1,500	118	110	105	85	1,500
Iron (mg)	245	247	260	327	8	109	110	116	145	18
Iodine (μg)	73	96	73	37	150	73	96	73	37	150
Selenium (μg)	192	185	152	139	55	192	185	152	139	55
Magnesium (mg)	136	133	139	170	400	176	172	179	220	310
Phosphorus (mg)	243	250	249	229	700	243	250	249	229	700
Zinc (mg)	105	99	101	100	11	144	137	139	137	8
LA, 18:2 ω-6 (g)	80	82	87	100	17	114	117	124	142	12
ALA, 18:3 ω-3 (g)	46	51	58	75	1.6	66	74	85	109	1.1

RDA (Recommended Dietary Allowance) and AMDR (Acceptable Macronutrient Distribution Range) established by IOM. All intakes are below the Tolerable Upper Intake Levels (ULs) established for vit B3 (35 mg), vit B6 (100 mg), vit B9 [folic acid] (1,000 μg), vit C (2,000 mg), vit D (100 μg), vit E (1,000 mg), calcium (2,500 mg for 19–50 y; 2,000 mg for ≥51 y), iron (45 mg), iodine (1,100 μg), selenium (400 μg), magnesium (350 mg from supplements), phosphorus (4,000 mg), and zinc (40 mg). ¥ nutrients with Adequate Intake (AI) reference values. ^*^ Retinol (preformed Vit A). LA: linoleic acid: ALA: linolenic acid; AA: araquidonic acid.

The omnivorous, pesco-vegetarian and ovo-lacto-vegetarian patterns showed similar adequacy profiles, whereas the vegan pattern deviated only for a restricted set of micronutrients.

None of the four menus (diet groups) reached full (100%) coverage regarding the RDA for vitamins B₅ and vitamin D, and minerals including, iodine, or the essential fatty ALA, with some exceptions. Essential fatty acid requirements were met in vegan men regarding LA, only. Intake of ALA was under the threshold given by the RDA in all diet groups. By contrast, in women, LA requirements were met across the diet groups, whereas intakes of ALA complied with the recommendations among the vegans. These potential differences between men and women could be attributed to the 2,000 kcal diet menu fixed for both sexes.

When comparing the menus, the vegan menu showed higher coverage for vitamin B₅ (30%), and LA and ALA (100 and 75%, respectively). The pesco-vegetarian menu had marked gains for vitamin D (25% of RDA) and iodine (96%). And the ovo-lacto-vegetarian menu showed intermediate increases for LA (87%) and ALA (58%). Moreover, the lowest adequacy values were observed for iodine and vitamin D in the ovo-lacto (73% and 6%) and vegan (37% and 0%), and for vitamin B_12_ in the vegan (13%) menus.

Sodium coverage was slightly lower in the pesco-vegetarian (126–110%) and ovo-lacto- vegetarian (122–105%) patterns than in the omnivorous group (136–118%), with the vegan pattern showing the lowest range (98–85%). Conversely, iron coverage was very high for all menus and maximal in the vegan diet (327%), followed by the ovo-lacto (260%), pesco (247%) and omnivorous (245%) groups. The vegan menu also provided the greatest coverage for vitamin B_1_ (148–196%), vitamin B_2_ (218–226%) and vitamin B_9_ (179%).

With regard to compliance with the NRV (vitamins: A, D, C, E, B_1_, B_2_, B_3_, B_6_, B_9_, B_12_; minerals: phosphorus, iron, magnesium, zinc), reference values were likewise not met for vitamin D, and for vitamin B_12_ in the vegan diet (data not shown).

Nutrient intake as a percentage of total diet by the type of diet is shown in Supplementary Table S6. Overall, PB foods were the major contributors to the dietary nutrient intake (iron, calcium, etc.) in PBDs.

### 3.4 Environmental impact assessment of the menus

**Figure 1:**
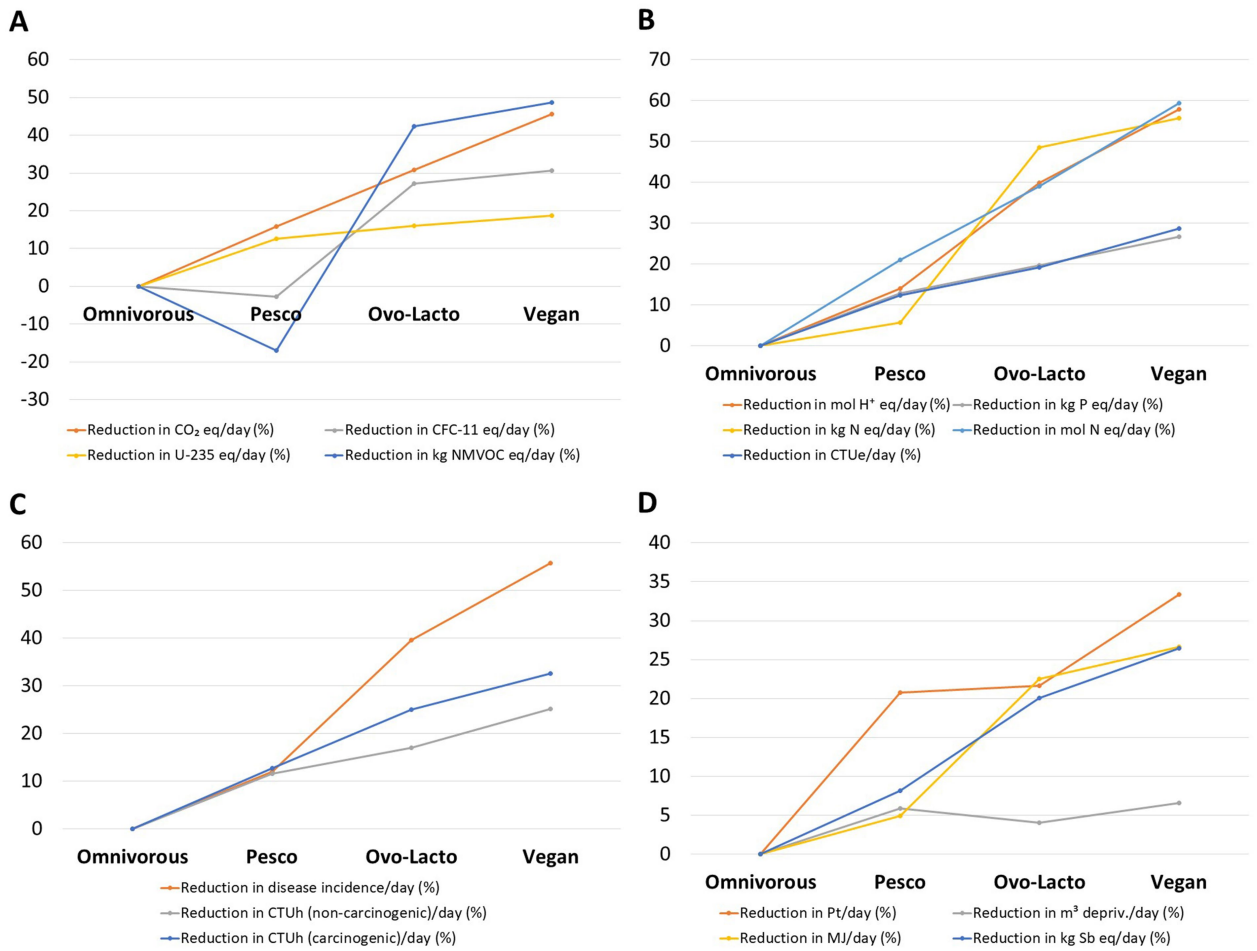
Relative reductions (%) in environmental and health footprint indicators across dietary patterns compared with an omnivorous baseline. Values are expressed as percentage decreases per day for pesco-vegetarian, ovo-lacto-vegetarian, and vegan menus. Indicators include climate change and emissions **(A)**, ecosystem quality **(B)**, human toxicity **(C)**, and resources **(D)**. Negative values indicate higher impacts than the omnivorous diet for that specific indicator.

Environmental assessment revealed a clear gradient across the four dietary patterns (Figure 1; Supplementary Table S7). Mean cradle-to-home greenhouse gas emissions (CO₂e) declined stepwise from the omnivorous menu (3.8 ± 9.1 kg/day) through the pesco-vegetarian (3.2 ± 9.1 kg) and ovo-lacto-vegetarian plans (2.6 ± 5.2 kg), reaching the lowest value in the vegan menu (2.1 ± 4.6 kg). The same ranking was observed for deprived water use (10.2 ± 2.1 m^3^ to 9.5 ± 2.1 m^3^) and agricultural land occupation (226 ± 84.1 Pt/NW of product to 151 ± 58.4 Pt/NW of product). There were statistically significant differences between the dietary patterns for two indicators (CO₂e, *p* = 0.001; land, *p* = 0.02). Each animal food substitution by a PB alternative, first replacing meat with fish, then with legumes, dairy and eggs, and finally adopting a fully vegan composition, yielded a statistically meaningful reduction in environmental footprint, with the pesco-vegetarian and vegan plan cutting gas emissions, deprived water and land demand by 15 to 46% (Figure 1A), 4.0 to 6.6% and 21 to 33% (Figure 1D), respectively, relative to the omnivorous diet.

Among other relevant indicators, several exhibited reductions following a similar trend of about 50%, including kg NMVOC eq/day (−17 to 49%; *p* = 0.01), disease incidence/day (12 to 56%; *p* = 0.001), mol H^+^ eq/day (14 to 58%; *p* = 0.001), kg N eq/day (5.7 to 56%; *p* = 0.01), and mol N eq/day (21 to 59%; *p* = 0.0005). Notably, the pesco-vegetarian diet was associated with increases in CFC-11 eq/day and NMVOC eq/day.

## 4 Discussion

The present study assessed the nutrient profile and the environmental footprint of a 2,000 kcal omnivorous 7-day menu modelled on the Spanish dietary guidelines reflecting a Mediterranean diet, and of three equivalent PBDs where animal-based foods were replaced by PB foods according to specific PBD guidelines. The three PB menus were modelled for a pesco-vegetarian, ovo-lacto-vegetarian and vegan diet (22, 26). To our knowledge, this is the first study to apply dietary guidelines to simulate both PB and non-PBDs, in order to evaluate and compare, under isocaloric conditions, their environmental impact and nutritional adequacy. Through an integrated analysis of their alignment with nutritional goals, dietary recommendations and a comprehensive set of environmental footprint indicators, this study provides novel data on the sustainability and nutritional balance of PBDs of different types.

Together, the four menu plans provided the same amount of energy and were balanced with regard to the macronutrient composition to facilitate nutritional comparisons. Only the intake of fat and fibre deviated to some extent in the vegan diet due to the nutritional composition of foods defining this dietary pattern. These comparisons revealed similar intakes of various nutrients and compliance with dietary recommendations and nutritional intake, except for vitamin D, vitamin B_12_, iodine and certain PUFAs. The most meaningful difference between the dietary patterns were noted with regard to vitamin B_12_, whose intake met the recommended intakes in all PBDs, except in the vegan diet. Furthermore, this study demonstrates that dietary transition towards a PBD results in an up to 46% reduced environmental impact.

The Mediterranean diet was considered as the reference dietary model, considering that this is a predominantly PB and health-promoting dietary pattern. In fact, this model defines high consumption of whole grain cereals (at least 1 serving/day) and considerable amounts of fruits, vegetables (3–4 servings/each) and other PB foods. As a consequence, this dietary pattern consequently resulted in elevated fiber intake, low SFA intake, and aligned well with nutritional recommendations. The PBDs models derived from it showed similar nutrient composition and could be regarded as nutritionally equivalent and healthy. Thus, our results support that a similar outcome can be achieved by replacing meat and meat products with fish and shellfish, eggs, milk, and dairy products. Moreover, the pesco-vegetarian diet could be considered as a dietary pattern close to the Mediterranean diet, followed by the ovo-lacto-vegetarian diet. Importantly, the vegan diet model showed a more distinctive profile due to the avoidance of animal-based foods, with the highest intake of fibre, the lowest intake of SFA and cholesterol, and the highest intake of PUFAs. Thus, the lipid profile of this diet could be considered more favorable. In contrast, this diet model also showed insufficient intakes of nutrients including vitamin D, vitamin B_12_ and iodine. The most striking differences of the four dietary models are described below.

### 4.1 Protein requirements and concerns in plant-based menus

Protein requirements were met in every menu plan or diet model. A previous Belgian cross-sectional survey (*n* = 1,475) reported the lowest energy and protein intakes in vegans. We removed this energy-intake bias by comparing isocaloric menus when calculating overall nutrient density. However, it was not possible to explore whether there are differences by the amino acid profile of the four menus (diet groups) since the BEDCA food composition table lacks this data. In a large-scale study on the amino-acid content of 2,335 foods it was shown that the amino-acid content vary two- to six-fold across the food supply, with PB dietary patterns clustering at the lower extreme for lysine, threonine, tryptophan and methionine while remaining broadly comparable for most other amino acids (45). Thus, it can be assumed that proteins provided in the PBD menus are low in these amino acids. In fact, within the EPIC-Oxford study, a biomarker analyses found circulating lysine, methionine, leucine, valine and tryptophan to be 6–13% lower in long-term vegans than in meat-eaters, with vegetarians exhibiting intermediate levels (46).

### 4.2 Micronutrient shortfalls and critical nutrients

Micronutrient shortfalls were limited to a small set of nutrients: vitamins D and iodine (except among pesco-vegetarians) in every diet model, with an additional likely deficit of vitamin B₁₂ in the vegan diet. These results agree with findings from large observational studies, as those included in a recent narrative review (5) and umbrella review (20), which concluded that PBDs are typically low in vitamins B₁₂ and D, calcium and iodine, but high in vitamins B_9_, C and E, phosphorus and magnesium. Our dietary models reproduce in part this pattern: the vegan model provides limited amounts of vitamin B₁₂, vitamin D and iodine but excels in vitamins B_9_, C, E and magnesium, calcium and phosphorus. While some studies have argued that there are several critical nutrients in the PBDs (5), the present modelling study does not support this. These data must be interpreted with caution, however, because the bioavailability of nutrients can differ substantially between plant and animal food sources. No menu exceeded the ULs.

#### 4.2.1 Calcium

All modelled diet plans exceeded the threshold intakes of calcium set for adults by the EFSA (adequate intake: AI = 950 mg/day), the WHO reference intake (reference intake: RNI = 1,000 mg/day), and those given by the IOM (DRIs = 1,000–1,200 mg/d). Among the PBDs, the ovo-lacto-vegetarian pattern scored highest because it included milk and dairy products, which is the main dietary source of this nutrient (51–53% of Ca in the diet; Supplementary Table S6). Other important contributors were vegetables, legumes, nuts (46–49%), whereas fish and other animal foods accounted for only 1–2% of dietary Ca, based on FCT data and our menu models (27). Remarkably, the vegan menu resulted in a calcium intake similar to that of the omnivorous model, a result that agrees with some studies on this topic (18, 47) but diverges from others (2, 47), possibly due to the lack of isocaloric comparisons in these earlier studies. For instance, a meta-analysis of 74 studies reported that vegans consume on average 30% less calcium than omnivores (729 mg/day vs. 993 mg/day) (18). Some small cross-sectional studies have also reported differences in calcium status between vegans and omnivores (47). These differences have been related with potential impacts on bone health. As such, the EPIC-Oxford cohort reported a 40% higher fracture risk in vegans (48), whereas the Adventist Health Study-2 found no excess fractures when calcium and vitamin D intakes were adequate (49). Other modelling studies addressing the nutritional balance of the EAT-Lancet diet as a proxy for a PBD, in comparison to other healthy dietary guidelines, have encountered inadequate amounts of calcium (50, 51). This lower calcium intake observed in the EAT-Lancet diet may be explained by the limited allowance of dairy products (~250 g/day), which are the primary source of calcium (52). In our modelling study, the Spanish dietary guidelines seem to overcome this limitation thanks to the higher recommended intake of dairy foods. In fact, the EAT-Lancet Commission also proposed recommendations to address current shortfalls, including considerations of nutrient bioavailability and specific needs across genders and population groups, among others (53).

The UVE recommends two daily servings of calcium-rich foods such as fortified PB beverages or yogurts, cruciferous and low-oxalate leafy vegetables, tofu, nuts and seeds. Calcium absorption efficiency, however, is highly variable: 5% from oxalate-rich greens (spinach and chard); 20–25% from legumes, nuts and tahini; 32% from soy products and calcium-carbonate-fortified soy milks — equivalent to cow’s milk —, and > 50% in certain green leafy vegetables (e.g., kale), whereas absorption of tricalcium-phosphate-fortified drinks are 20% lower (52). Phytate and oxalate chelation hinder the absorption of calcium, but soaking, sprouting or fermenting markedly improve its bio-availability (52). These culinary methods are therefore key to enhance the calcium bioavailability in a PBD. Importantly, PBDs also generate a lower renal acid load than meat-rich diets, reducing urinary calcium excretion and enhancing the net calcium balance (52). Our study also shows that the Ca/P ratio of the four diet groups could affect the absorption of calcium in PBDs, despite this ratio being adequate in terms of dietary intake of the two nutrients. Furthermore, the fractional absorption of calcium is expected to be lower in PBDs, likely due to the aforementioned chelation effects (54). As outlined previously, this potential reduced absorption of calcium could affect bone health in individuals following strict PBDs (47, 55).

In terms of bioavailability of calcium, gastric acid first solubilises calcium and uptake along the gut proceeds via a vitamin-D-regulated pathway (55). Vitamin D levels are also key to optimize the absorption of this nutrient. Luminal factors also have an influence on the absorptive efficiency of calcium; for instance, the presence of certain amino acids such as leucine, proline, hydroxyproline, isoleucine, alanine, lysine and small peptides may stimulate Ca^2+^ transport (55). Conversely, high sulfur-amino-acid loads increase urinary calcium losses, while increasing absorption of zinc (52). Besides, it has been noted that calcium is absorbed more effectively when the food matrix is liquid, which supports the use of calcium-fortified PB beverages. Soy drink is particularly relevant in this regard: its essential amino-acid scores for threonine, leucine and lysine meet 92.7%, 90.8%, and 77.4% of requirement values. Thus, soy drink may help optimize calcium absorption, especially when fortified with calcium (52, 56).

#### 4.2.2 Iron

The vegan menu model supplied the highest iron intake (26.1 mg/day) compared with the omnivorous (19.6 mg/day), pesco-vegetarian (19.8 mg/day) and ovo-lacto-vegetarian (20.8 mg/day) models. Similar intake hierarchies have been reported in other studies, where vegan diets provided the highest iron intake. Meat, fish, cereals, nuts and eggs, are the richest food sources of this nutrient (27). A greater intake of these foods, however, does not ensure an optimal iron status because plant foods contain only non-heme iron, whose fractional absorption (5–10%) is considerably lower than that of heme-iron (15–30%) contained in animal foods, due to its ferric coordination chemistry and low solubility. Regarding non-PBDs, the heme-iron content of meat varies considerably (20–70%) depending on the meat type (57), contributing significantly to total iron intake in omnivorous diets and influencing overall iron bioavailability. In this regard, our modeled diets show that 89% of total iron intake in the omnivorous pattern was derived from non-heme sources, while heme iron contributed 6.2% (Supplementary Table S6). In the pesco- and ovo-lacto-vegetarian diets, non-heme iron accounted for 93–95% of total intake, with heme iron contributing 2.2% in the pesco model only, and 4.3–4.5% originating from eggs and dairy products. In the vegan diet, iron intake was only supplied as non-heme iron. Our findings are consistent with previous reports of heme iron intake in omnivorous, vegetarian and vegan diets (58).

Bioavailability can, however, be enhanced when meals contain adequate vitamin C and others small organic acids (citric, malic, lactic) that reduce Fe^3+^ to Fe^2+^ and form soluble chelates (59). Since PBDs are rich in vitamin C, it can be assumed that the absorption of non-heme iron is facilitated (58). Traditional processing methods (soaking, etc.) further improve uptake by hydrolysing phytic acid, the main chelator of divalent metals in PBDs rich in whole grain cereals and legumes. Soybean ferritin represents an additional exception, with an intermediate absorption of 22–34% (52).

Because median fractional iron absorption from PBDs is only 10% (vs. 18% in mixed diets), the IOM applies a 1.8 correction factor to the RDA reference values; this raises daily requirements from 8 mg to 14 mg for adult men and from 18 mg to 32 mg for pre-menopausal women. In our simulations, the vegan menu model met the male target but fell slightly short of the female requirement (34).

Epidemiological evidence indicates that lower bioavailability does not necessarily lead to higher anaemia rates. The clinic-based study of 1,340 Brazilian adults found no excess of true iron deficiency in healthy vegetarians or vegans except among menstruating women (60). Likewise, a meta-analysis of 27 studies showed significantly lower ferritin concentrations in vegans/vegetarians (pooled mean difference: 34 μg/L) without a higher prevalence of iron-deficiency anaemia, implying that roughly half of the additional iron typically consumed in PBDs is not retained (61). However, a recent study found that vegans and vegetarians have undergone a metabolic adaptation that allows them to absorb non-heme iron more efficiently than omnivores (62).

#### 4.2.3 Iodine

The intake of iodine did not reach the adult RDA of 150 μg/day in any menu. Predicted intakes were 119 ± 16 μg in the Mediterranean pattern (73% of RDA), 143 ± 61 μg in the pesco-vegetarian (96%), 109 ± 19 μg in the ovo-lacto-vegetarian (73%) and only 55 ± 21 μg in the vegan plan (37%). The shortfall in the vegan menu could reflect the exclusion of the richest dietary iodine source; mainly marine products, eggs and milk (27). However, it should be noted that iodized salt contribution was not quantified in any of the dietary patterns analyzed in our study. Given the widespread use of iodized salt, its consumption is expected to compensate for the apparent iodine shortfalls across all menus, particularly in the vegan pattern where natural dietary sources of iodine are more limited (63).

Iodine absorbed as free iodide (the form present in iodized salt, dairy, fish and eggs) is >90% bioavailable; excess is rapidly excreted in urine and only 10% is retained for thyroid-hormone synthesis. By contrast, part of the iodine bound to polysaccharides in certain brown seaweeds has a relatively low and highly variable absorption despite its high iodine content (1.5–2.4 mg/g) (20, 64). Isoflavones from soy and glucosinolates from cruciferous vegetables can further inhibit thyroidal uptake of iodide and accentuate the need for a sufficient intake (63). Epidemiological data confirm that 60–70% of vegetarians and vegans fall below 150 μg/day when they neither supplement nor consume seaweed, with minimal intakes of 17 μg/day reported in UK vegans (20).

Three practical strategies could enhance reliable iodine supply in PBDs: (1) use of iodized salt (63); (2) consumption of fortified foods such as PB milks or breads providing 100–150 μg/serving, which have been shown to raise intakes in vegetarians and vegans (20); and (3) occasional inclusion of iodine-rich seaweeds (65).

#### 4.2.4 Phosphorus

The main food sources of phosphorus are milk and dairy products, as well as grains (27). Phosphorus requirements (RDA = 700 mg/day) were met in every modelled menu, although the vegan pattern provided the smallest amount. This mineral was predominantly supplied by PB foods, accounting for 61–69% of total intake in the omnivorous, pesco-, and ovo-lacto-vegetarian diets (Supplementary Table S6). However, there might be substantial differences between the diet patterns because absorption of this nutrient depends on the chemical form of this mineral; animal foods contain hydrolysable organic phosphates, whereas plant foods store phosphorus mainly as phytic acid (phytate), of which <50% is absorbed in humans lacking intestinal phytase (54).

Intervention studies confirm that, at equal total intake, vegetarian diets have lower levels of serum phosphate and reduced urinary excretion than omnivorous diets (66), reflecting the smaller absorbable fraction in PBDs. In agreement with this, it has been shown that the usable phosphorus load of predominantly PBDs falls by 40%, whereas phosphorus from additive phosphates in ultra-processed products is absorbed almost entirely (67).

The vegan menu modelled in this study, intentionally minimised ultra-processed substitutes, to keep the definition of a healthy diet. The PB alternatives were soy milk and soy yogurt, and texturized vegetable proteins, and thus, may contribute little to highly absorbable phosphate. However, this does not imply any insufficiency as phosphorus intake is well above recommended levels. In fact, the current RDA values assume 65% absorption in a mixed diet, yielding 420 mg of usable phosphate. If a diet supplied phosphorus almost exclusively as phytate, with only 30–40% absorption, roughly 1,200 mg/day (1.6 × 700 mg) would be needed to deliver the same absorbed dose. All PB models in this study provided ≥ 1,500 mg of this nutrient, ruling out any risk of phosphorus deficiency despite the lower bioavailability from PB food sources (54). Also, culinary practices, such as soaking and fermentation, activate endogenous or microbial phytases that can raise phosphorus (and iron, zinc) bioavailability (68).

#### 4.2.5 Cobalamin (B_12_)

In our menus, omnivorous patterns surpassed the RDA for vitamin B_12_ (≥ 2.4 μg/day) in adults. In contrast, the ovo-lacto- and pesco-vegetarian diets, while meeting the recommendations, are close to the lower limit of adequacy. Given the potential culinary losses of vitamin B_12_ (20–40%), its relative content in milk (0.5 μg/100 g), dairy products (4.2 μg/100 g), and eggs (2.5 μg/100 g) is reduced to approximately 0.4, 3.6, and 1.9 μg/100 g, respectively (28). Also, the dose-dependent fractional absorption (approximately 40–60% at physiological doses), limits the total amount of bioavailable B_12_, which may fall below 2 μg/day.

Studies in vegetarian cohorts have shown that serum B_12_ can remain within the reference range for several years, even if tissue depletion progresses. Hence, insufficient dietary intake of this vitamin, while not causing immediate health effects, can lead to megaloblastic anaemia or neuropathy by 2–5 years (52). Current consensus, therefore, recommends systematic supplementation through cyanocobalamin (heat-stable, inexpensive) or methylcobalamin (bio-active but less stable) at 25–250 μg/day, or highly fortified foods for all vegetarians, but especially for vegans (69).

Vitamin B_12_, which is only contained in animal foods, is released from food proteins in the stomach, bound first to salivary–gastric haptocorrin, transferred to intrinsic factor (IF-B_12_) in the duodenum, and finally absorbed in the distal ileum via the cubilin receptor. Lysosomal export into the enterocyte cytosol depends on ABCD4 and its chaperone LMBD1. Once in the cytosol, vitamin B_12_ is processed by the MMACHC protein, to be converted in its active forms: methylcobalamin in the cytosol and adenosylcobalamin in the mitochondria. In blood, vitamin B_12_ is transported bound to two proteins: transcobalamin II, which delivers active cobalamin to body tissues, and hepatocorrin, which binds the majority of circulating B_12_ in a non-functional storage form (70–73). This multi-step pathway explains why absorption efficiency saturates at doses above 2 μg (bioavailable vitamin B_12_). In individuals with limited absorption capacity of vitamin B_12_ or those following vegan diets, passive diffusion becomes relevant. This mechanism does not require the IF or receptors to be absorbed, but its diffusion into the gastrointestinal tract is of very low efficiency. In fact, only 1% of the ingested vitamin B_12_ is absorbed via passive diffusion. High oral doses of over 500 μg are needed to turn this mechanism efficient. Current guidelines of vitamin B_12_ supplementation in vegans (25–250 μg daily or ≥ 1,000 μg once weekly) are based on passive diffusion absorption (52).

Fortified foods can help vegetarians approach the RDA for vitamin B₁₂, but this is not supported for vegans. In fact, it has been shown that commercial PB beverages (milks) can deliver 2–5 μg B₁₂ per glass and are effective at maintaining serum B₁₂ when consumed daily (52, 71, 73). Yet intakes based solely on such products depend on strict, day-to-day compliance and can be highly variable: in the EPIC-Oxford cohort, 52% of vegans but only 7% of vegetarians had serum B₁₂ < 118 pmol/L despite access to varied vitamin B_12_-fortified foods (46). In our study, only a small amount of this vitamin was provided by the vegan diet, primarily through the consumption of fortified cereals.

Novel plant sources occasionally marketed to vegans require caution. Vitamin B₁₂ from duckweed (water lentil: *Lemna minor*) appears bioavailable, whereas in Spirulina, a supplement often consumed by vegans due to its high content of proteins, iron, and B vitamins, contain inactive vitamin B_12_ forms that may even compete for the transcobalamin transporter (71). Thus, while fortified PB milk alternatives and yeast are valuable adjuncts for vegetarian diets and convenient for public-health programs, in strict vegans they are complementary rather than sufficient, a point of particular concern during pregnancy, lactation and infancy (73). Supplements, therefore, remain the only dependable long-term strategy in the vegan diet.

#### 4.2.6 Vitamin D

Vitamin D dietary requirements were not met by any of the four diets, with the pesco-vegetarian diet being the only one reaching 25% coverage of the RDAs, thanks to a higher intake of fish (main dietary source). In contrast, the ovo-lacto-vegetarian and Mediterranean diet achieved less than 20% coverage, whereas the supply of this vitamin in the vegan diet was almost negligible. In our study, the most important source of vitamin D in the vegan diet was soy milk fortified with this nutrient. The difference in coverage between the omnivorous and pesco-vegetarian diets was due to the inclusion of one additional serving of fish in the pesco-vegetarian diet. Earlier studies on nutritional differences between the vegan and omnivore diets have reported similar findings (5, 74). In contrast, in a study from the UK using dietary data of 81 women, that analysed the variation in vitamin D intake by replacing animal-based foods with equivalent amounts of PB alternatives, it was concluded that Vitamin D intake did not substantially change between the baseline and post-replacement diet. Intakes of vitamin D were below 4 μg/day, this intake being close to ours (75).

Vitamin D exists in two main forms: D_2_ (ergocalciferol), the most bio-available form, found in fungi, and D_3_ (cholecalciferol), found in animal-based foods and produced in the skin via sun exposure. Therefore, PBDs, and more importantly the vegan diet, are likely to present lower levels of serum 25(OH)D. However, while dietary intake determines vitamin D status, the primary and most efficient source is cutaneous synthesis via sunlight (UVB exposure) (76). In fact, for most people, adequate sun exposure can meet or exceed daily requirements, even if dietary sources, especially in PBDs, are typically insufficient on their own. However, some studies have shown that the prevalence of vitamin D deficiency is higher in vegan than in omnivores (74, 77). Thus, ensuring adequate dietary vitamin D intake through diet becomes essential in PBDs. While some mushrooms exposed to UV light provide D_2_, the main dietary sources for vegans are fortified foods (such as PB milks) and supplements, preferably D_3_ from lichen (65). Therefore, supplementation with vitamin D may be necessary when following PBD patterns.

#### 4.2.7 Other minerals and vitamins

The intake of selenium, mainly provided by vegetables and grains including soya beans, also differed significantly between the four dietary models, with this difference being driven by the vegan diet. Nonetheless, selenium content in foods is rather inaccurate in food composition tables (78). Also, intake of this nutrient met the recommended levels across all groups, suggesting no relevant risk of deficiency. Finally, regarding sodium, while intake was also significantly lower in the vegan diet, absolute values remained within acceptable ranges in all dietary models.

Vitamins B_1_ and B_3_ also showed differing levels of intakes between the four dietary models (highest and lowest in the vegan diet, respectively), but both reached the RDA. For vitamin B_5_, there were no differences in intake between the four dietary groups, although none of them met the RDA. However, this result is not considered of concern, as specific data on vitamin B_5_ was often lacking in the BEDCA food composition database, which may have led to underestimation of the actual intake. Indeed, vitamin B_5_ deficiency is unlikely in well-nourished populations (79). All other vitamins in the dietary models showed similar intakes, in agreement with previous studies evaluating nutrient intakes in PBDs (5).

### 4.3 Fatty-acid profile: MUFA, PUFA and long-chain *ω*-3

Our modelled menus showed that none of the four menus reached the guideline of MUFA intake relative to total energy (E) intake (>20%). The estimated MUFA supply reached 32 g/day (14.4% of E) in the Mediterranean, pesco- and ovo-lacto- diet models and fell to 29 g/day (13.1% of E) in the vegan diet. However, since extra virgin olive oil was the primary fat source used in all models, the overall quality of fat intake can be considered adequate (30).

The intake of SFA also complied with dietary guidelines, remaining below the <7–8% of E threshold (as low as possible), and was even more favourable (halved) in the vegan diet. Consequently, the PUFA: SFA ratio reached 1.1 in the three animal-containing diet models but increased to 2.7 in the vegan model. Likewise, the combined (MUFA + PUFA): SFA ratio improved from 2.7 to 5.8.

Total PUFAs met the requirements (> 5% of E) in every pattern, ranging from 10% of E in the omnivorous and vegetarian diet model to 11.4% of E in the vegan one, likely because daily consumption of nuts, seed or soy should elevate the intake of LA. Indeed, LA intakes contributed to 6–7% of energy intake in the Mediterranean, pesco- and ovo-lacto-vegetarian models (reference value: 3% of E), and rose to 7.7% in the vegan diet (*p* = 0.034). When considering the ALA supply in terms of the LA: ALA ratio, all were ≤ 8:1 range regarded as cardio-protective (80). Our result is consistent with data of other studies showing LA: ALA ratios of 15–20 in vegans and vegetarians (80).

With regard to the intake of ALA, the recommendations were not fulfilled since all diet models were below the 1% of E target (30). Previous studies have also evidenced low intake levels of ALA in the Spanish population, even among those following a healthy diet (81). In particular, EPA intake was sub-optimal in our study. It reached 60 mg/day in the omnivorous model and 13 mg/day in the pesco-vegetarian diet, but only 1 mg/day in the ovo-lacto and vegan diets. DHA showed a similar decreasing gradient. Thus, none achieved the EFSA target of 250 mg/day for EPA + DHA (82), which agrees with findings of the EPIC–Oxford study that support that the plasma EPA + DHA status is lower in PBD groups despite up-regulated conversion from ALA (46, 72, 73). In fact, when *ω*-3 PUFAs are scarce, the dietary balance between LA and ALA gains importance because a lower LA: ALA ratio favors endogenous EPA synthesis from ALA (82). In any case, to reach the recommended levels of intake, several authors have claimed to consume two 100-g portions of oily fish weekly (83), or a daily algal-oil supplement, which has been specifically endorsed for vegetarians and vegans (84). However, the EFSA panel sets an adequate intake for ALA at 0.5% of E, based on the lowest estimated mean intakes of the various population groups from a number of European countries without deficiency signs, and does not endorse any specific ω-3/ω-6 ratio (82). In light of these considerations, to supply sufficient intakes of ALA, current dietary guidelines should account for the inclusion of oily fish, algae-based food and fortified foods.

### 4.4 Environmental-impact

The life-cycle model of our seven-day diet menus showed a notable reduction in climate change–related factors (Figure 1A), including CO₂ emissions, ozone depletion, ionizing radiation, and photochemical ozone formation, for both ovo-lacto-vegetarian and vegan diets. This gradient matches in the case of CO₂e value reported in other studies (a reduction of 35% for ovo-lacto-vegetarians and 50% for vegan diets) (52). Moreover, in an Italian study, vegan diets averaged 2.06 kg CO₂e/day, which is almost identical to our 2.07 kg, whereas omnivorous diets approached 3.6 kg CO₂e/day, similar to our 3.80 kg estimate (85). Climate change–related factors reductions are environmentally significant, as they imply a lower contribution to global warming, decreased stratospheric ozone loss, and diminished formation of secondary air pollutants that adversely affect air quality (2). In contrast, the pesco-vegetarian diet was associated with increases in ozone-related indicators, which can be explained by the high environmental costs of fish production and distribution. In particular, refrigeration, storage, and long-distance transport of seafood often involve emissions of refrigerants and volatile organic compounds, thereby offsetting some of the potential environmental gains of reducing meat consumption.

Additionally, our PB models, including the pesco-vegetarian diet, showed reductions in the percentage of indicators related to ecosystem quality (Figure 1B), human health (Figure 1C), and resources (Figure 1D). Within ecosystem quality, three indicators exhibited reductions > 50% in the vegan diet compared with the omnivorous baseline: acidification (mol H^+^ eq/day), marine eutrophication (kg N/day), and freshwater ecotoxicity (CTUe/day). Acidification and eutrophication processes directly impair soil fertility, aquatic biodiversity, and crop yields, while ecotoxicity reflects the potential accumulation of toxic substances in freshwater ecosystems, threatening both wildlife and human populations through the food chain. Closely linked to these ecosystem impacts are implications for human health. In this regard, we also observed a marked reduction in disease incidence per day, with decreases > 55% in the vegan model compared with the omnivorous baseline.

In terms of resource use, all PB diets, including the pesco-vegetarian model, showed a reduction in land use exceeding 20% compared with the omnivorous diet, results that are also consistent with previous studies (2, 86), while other authors have reported nearly double reductions of around 42% in land use (52). Consistent decreases in energy demand (MJ/day) as well as mineral and metal use (kg Sb eq/day) were observed only in the ovo-lacto-vegetarian and vegan models. No direct comparison with existing data is possible to corroborate this finding. By contrast, no substantial differences were found for water use, calculated as deprivation according to the AWARE method, with reductions of around 5% across all three PB dietary patterns. A previous review of 41 studies conducted worldwide that focused on the water footprint of dietary patterns, reported similar estimates of water use for “healthy” diets (87). Specifically, this review included two studies based on survey data and simulation models of the Mediterranean Diet, both of which showed that adherence to this dietary pattern results in water savings compared with Western diets (88, 89). In another recent diet simulation study, water demand for the adherence to the Spanish, Italian and American dietary guidelines was evaluated, with similar water use estimates being reported across the dietary patterns (90). While this study estimated the contribution of animal and PB food sources to the total water demand, it did not provide data on PB dietary patterns as a whole (for example, a vegan diet) as we did in our analysis.

Lower greenhouse gas-emission diets have been linked to 12–22% fewer premature deaths by 2030, driven mainly by reduced red-meat intake and higher fruit-and-vegetable consumption (2). Thus, the footprints calculated in the present study corroborates that replacement of animal foods, especially ruminant meat, by PB foods, offers a robust, quantifiable contribution toward the climate and biodiversity targets for the year 2030.

## 5 Conclusion

This comparative study of four isocaloric 2,000 kcal diet models (Mediterranean, pesco-vegetarian, ovo-lacto-vegetarian and vegan) confirmed that all dietary patterns can meet protein, energy and most micronutrient targets when based on the dietary guidelines of a sustainable and healthy diet. Nutritional inadequacy risks were specific to iodine and to vitamins including D and B₁₂ in the vegan diet. The latter emphasizes the need of systematic fortification or supplementation plus periodic monitoring of vitamin B_12_. Routine consumption of fortified PB beverages or supplements is also recommendable in PBDs to support both vitamin D status when sunlight exposure is limited, and adequate calcium intake. Although iron provision exceeded the reference intakes, vitamin-C-rich foods and iron-fortified foods should be considered to enhance the absorption of non-heme iron in the PBD. Other nutrients, such as *ω*-3 PUFAs, also deserve consideration in PBDs.

Taken together, this study supports the feasibility of nutritionally adequate, environmentally sustainable PBDs, which substantially reduce greenhouse gas emissions, improve ecosystem quality, and lower the release of toxic particles harmful to human health. Nevertheless, it is important to note that effective implementation of PBDs require proactive strategies to ensure adequate intakes of essential nutrients, e.g., vitamin B₁₂, vitamin D, iodine, and long-chain ω-3 PUFA.

## Data Availability

The raw data supporting the conclusions of this article will be made available by the authors, without undue reservation.
