# Supplementary material for: Nutrient adequacy and environmental foot-print of Mediterranean, pesco-, ovo-lacto-, and vegan menus: a modelling study
Source: Front Nutr. 2025 Nov 11;12:1681512. doi: 10.3389/fnut.2025.1681512 (PMC12644066; doi:10.3389/fnut.2025.1681512)
Supplement: Supplementary file 1 [file Table_1.DOCX]

Supplementary Material

Comparative Nutritional Adequacy of Simulated Omnivorous, Vegetarian, and Vegan Diets: A 7-Day Menu Analysis Based on Dietary Guideline

# Supplementary Tables

**Supplementary Table 1.** SENC Recommendations for the Spanish Adult Population (2018).

**Supplementary Table 2.** Compliance of the 7-day menu plans with the SENC dietary guidelines and UVE recommendations.

**Supplementary Table 3.** Compliance of the 7-day menu plan with the Nutritional Goals set by the SENC.

**Supplementary Table 4.** Nutritional adequacy in adult (31-50 years) men and women (% of RDA) presented in the 7-day isocaloric menu (2,000 kcals) for each diet.

**Supplementary Table 5.** Nutritional adequacy in adult (51-70 years) men and women (% of RDA) presented in the 7-day isocaloric menu (2,000 kcals) for each diet.

**Supplementary Table 6.** Nutrient percentage provided from plant, animal, or daily/eggs foods-origin in omnivorous, pesco-, and ovo-lacto-vegetarian diets.

**Supplementary Table 7.** Total environmental fingerprint by type of diet (omnivorous, pesco-, and ovo-lacto-vegetarian diet).

**Supplementary Table 1.** SENC Recommendations for the Spanish Adult Population (2018).

| Food Group | Recommended Frequency | Weight of Each Serving (Raw) | Homemade Measurements |
| --- | --- | --- | --- |
| Milk and dairy products | 2-4 servings/day | 200-250 mL milk  200-250 g yogurt  40-60 g cured cheese  80-125 g fresh cheese | 1 glass of milk  2 yogurts  2-3 slices of cheese  1 individual serving |
| Bread, cereals, whole grains, rice, pasta, potatoes | 4-6 servings/day (increase whole grain varieties) | 40-60 g bread  60-80 g pasta, rice  150-200 g potatoes | 3-4 slices or a roll  2 handfuls or 1 plate of cooked rice  1 large or 2 small potatoes |
| Vegetables | At least 2 servings/day | 150-200 g | 1 plate of mixed salad  1 plate of cooked vegetables  1 large tomato, 2 carrots |
| Fruits | At least 3 servings/day | 120-200 g | 1 medium piece  1 cup of cherries  2 slices of melon |
| Olive oil | 3-6 servings/day | 10 mL | 1 tablespoon |
| Legumes | 2-4 servings/week | 60-80 g | 2 handfuls or 1 plate of cooked legumes |
| Nuts | 3-7 servings/week | 20-30 g | 1 small handful or 18-20 hazelnuts |
| Fish and seafood | 3-4 servings/week | 125-150 g | 1 small fillet |
| Lean meats and poultry | 3-4 servings/week (stagger their consumption) | 100-125 g | 1 small steak  1 quarter of chicken or rabbit |
| Eggs | 3-4 servings/week | Medium (53-63 g) | 1 egg |

**Supplementary Table 2.** Compliance of the 7-day menu plans with the SENC dietary guidelines and UVE recommendations.

| Food Group | Recommendations SENC | Recommendations UVE | Omnivore | Pesco-vegetarian | Ovo-lacto-vegetarian | Vegan |
| --- | --- | --- | --- | --- | --- | --- |
| Milk and derivatives | 2-4 servings/day |  | 2-3 servings/day | 2-3 servings/day | 2-3 servings/day | - |
| Bread, cereals, rice, pasta and potatoes | 4-6 servings/day |  | 4-6 servings/day | 4-6 servings/day | 4-6 servings/day | 4-6 servings/day |
| Vegetables | at least 2 servings/day |  | 3-4 servings/day | 3-4 servings/day | 3-4 servings/day | 3-4 servings/day |
| Fruits | at least 3 servings/day |  | 5 servings/day | 5 servings/day | 5 servings/day | 5 servings/day |
| Olive oil | 3-6 servings/day |  | 3 servings/day | 3 servings/day | 3 servings/day | 3 servings/day |
| Legumes | 2-4 servings/week |  | 2-4 servings/week |  |  |  |
| Nuts | 3-7 servings/week |  | 7 servings/week | 4 servings/week | 7 servings/week | 7 servings/week |
| Fish and seafood | 3-4 servings/week |  | 4 servings/week | - | - | - |
| Lean meats | 3-4 servings/week |  | 3 servings/week | 4 servings/week | - | - |
| Eggs | 3-4 servings/week |  | 4 servings/week | 4 servings/week | 4 servings/week | - |
| Legumes, soy and derivatives or seitan |  | 1-4 servings/day |  | 1-2 servings/day | 1-2 servings/day | 1-2 servings/day |
| Seeds |  | 1-2 servings/day |  | 2 servings/week | 2 servings/week | 2 servings/week |

The grey shading shows the food groups included in the SENC Dietary Guidelines and their recommendations; where grey shading is not indicated, the food groups included in UVE and their recommendations are shown.

**Supplementary Table 3.** Compliance of the 7-day menu plan with the Nutritional Goals (NGs) set by the SENC.

| Indices | Reference | Omnivore Mediterranean | Pesco-vegetarian | Ovo-lacto-vegetarian | Vegan |
| --- | --- | --- | --- | --- | --- |
| MUFA (% of E) | 20 | 14 | 14 | 14 | 13 |
| PUFA (% of E) | 5 | 10 | 10 | 10 | 11 |
| SFA (% of E) | < 7-8 | 8 | 8 | 8 | 4 |
| Fiber (g) | > 28 | 46 | 45 | 47 | 56 |
| PUFA/SFA | ≥ 0.5 | 1.2 | 1.2 | 1.2 | 2.5 |
| (PUFA + MUFA)/SFA | ≥ 2 | 3 | 3 | 3 | 5.5 |
| w-3 (ALA) (% of E) | 1-2 | 0.4 | 0.4 | 0.4 | 0.4 |
| w-6 (LA) (% of E) | 3 | 7 | 7 | 6 | 8 |
| w-6/w-3 ratio | 4/1 – 5/1 | 15/1 | 16/1 | 14/1 | 16/1 |
| Cholesterol (mg) | < 300 | 213 | 211 | 174 | 5 |
| Ca/P ratio | 1.3/1 | 1/1.5 | 1/1.4 | 1/1.3 | 1/1.4 |
| Fruits (g/d) | > 300-400 | 750 | 750 | 750 | 750 |
| Vegetables (g/d) | > 250-300 | 450-600 | 450-600 | 450-600 | 450-600 |

Cells shaded in grey indicate non-compliance.

SFA: saturated fatty acids; MUFA: monounsaturated fatty acids; PUFA: polyunsatured fatty acids

LA: linoleic acid: ALA: linolenic acid; AA: araquidonic acid; EPA: eicosapentanoic acid; DHA: docosahexaenoic acid

**Supplementary Table 4.** Nutritional adequacy in adult (31-50 years) men and women (% of RDA) presented in the 7-day isocaloric menu (2,000 kcals) for each diet.

|  | Men | | | | | Women | | | | |
| --- | --- | --- | --- | --- | --- | --- | --- | --- | --- | --- |
| Nutrient | **Omnivore** | **Pesco-Vegetarian** | **Ovo-lacto-Vegetarian** | **Vegan** | **RDA** | **Omnivore** | **Pesco-Vegetarian** | **Ovo-lacto-Vegetarian** | **Vegan** | **RDA** |
| Energy (kcal) | 107 | 104 | 106 | 110 | 2000 | 107 | 104 | 106 | 110 | 2000 |
| Protein (g) | 164 | 157 | 158 | 163 | 56 | 200 | 192 | 193 | 199 | 46 |
| Total carbs (g) | 202 | 193 | 197 | 226 | 130 | 202 | 193 | 197 | 226 | 130 |
| Fibre (g) | 120 | 119 | 123 | 148 | 38 | 183 | 181 | 186 | 225 | 25 |
| Vit B₁ (mg) | 158 | 148 | 149 | 180 | 1.2 | 173 | 162 | 163 | 196 | 1.1 |
| Vit B₂ (mg) | 168 | 162 | 164 | 185 | 1.3 | 199 | 191 | 194 | 218 | 1.1 |
| Vit B₃ (mg NE) | 180 | 166 | 143 | 135 | 16 | 205 | 190 | 163 | 154 | 14 |
| Vit B₅ (mg) ¥ | 23 | 23 | 23 | 30 | 5 | 23 | 23 | 23 | 30 | 5 |
| Vit B₆ (mg) | 254 | 247 | 238 | 243 | 1.3 | 254 | 247 | 238 | 243 | 1.3 |
| Vit B₉ (µg) | 148 | 149 | 152 | 179 | 400 | 148 | 149 | 152 | 179 | 400 |
| Vit B₁₂ (µg) | 152 | 180 | 121 | 13 | 2.4 | 152 | 180 | 121 | 13 | 2.4 |
| Vit C (mg) | 484 | 485 | 485 | 480 | 90 | 580 | 582 | 582 | 577 | 75 |
| Vit D (µg) | 19 | 25 | 6 | 0 | 15 | 19 | 25 | 6 | 0 | 15 |
| Vit E (mg α-TE) | 112 | 113 | 111 | 124 | 15 | 112 | 113 | 111 | 124 | 15 |
| Vit A (µg)* | 160 | 163 | 163 | 148 | 900 | 205 | 210 | 209 | 191 | 700 |
| Potassium (mg) ¥ | 155 | 154 | 154 | 168 | 3400 | 203 | 202 | 201 | 220 | 2600 |
| Calcium (mg) | 116 | 126 | 131 | 119 | 1000 | 116 | 126 | 131 | 119 | 1000 |
| Sodium (mg) ¥ | 118 | 110 | 105 | 85 | 1500 | 118 | 110 | 105 | 85 | 1500 |
| Iron (mg) | 245 | 247 | 260 | 327 | 8 | 109 | 110 | 116 | 145 | 18 |
| Iodine (µg) | 73 | 96 | 73 | 37 | 150 | 73 | 96 | 73 | 37 | 150 |
| Selenium (µg) | 192 | 185 | 152 | 139 | 55 | 192 | 185 | 152 | 139 | 55 |
| Magnesium (mg) | 130 | 127 | 132 | 162 | 420 | 170 | 167 | 174 | 213 | 320 |
| Phosphorus (mg) | 243 | 250 | 249 | 229 | 700 | 243 | 250 | 249 | 229 | 700 |
| Zinc (mg) | 105 | 99 | 101 | 100 | 11 | 144 | 137 | 139 | 137 | 8 |
| LA, 18:2 n-6 (g) | 80 | 82 | 87 | 100 | 17 | 114 | 117 | 124 | 142 | 12 |
| ALA, 18:3 n-3 (g) | 46 | 51 | 58 | 75 | 1.6 | 66 | 74 | 85 | 109 | 1.1 |

RDA (Recommended Dietary Allowance) and AMDR (Acceptable Macronutrient Distribution Range) stablished by IOM.

¥ nutrients with Adequate Intake (AI) reference values.

* Retinol (preformed Vit A).

LA: linoleic acid: ALA: linolenic acid

**Supplementary Table 5.** Nutritional adequacy in adult (51-70 years) men and women (% of RDA) presented in the 7-day isocaloric menu (2,000 kcals) for each diet.

|  | Men | | | | | Women | | | | |
| --- | --- | --- | --- | --- | --- | --- | --- | --- | --- | --- |
| Nutrient | **Omnivore** | **Pesco-Vegetarian** | **Ovo-lacto-Vegetarian** | **Vegan** | **RDA** | **Omnivore** | **Pesco-Vegetarian** | **Ovo-lacto-Vegetarian** | **Vegan** | **RDA** |
| Energy (kcal) | 107 | 104 | 106 | 110 | 2000 | 107 | 104 | 106 | 110 | 2000 |
| Protein (g) | 164 | 157 | 158 | 163 | 56 | 200 | 192 | 193 | 199 | 46 |
| Total carbs (g) | 202 | 193 | 197 | 226 | 130 | 202 | 193 | 197 | 226 | 130 |
| Fibre (g) | 153 | 151 | 155 | 188 | 30 | 183 | 181 | 186 | 225 | 25 |
| Vit B₁ (mg) | 158 | 148 | 149 | 180 | 1.2 | 173 | 162 | 163 | 196 | 1.1 |
| Vit B₂ (mg) | 168 | 162 | 164 | 185 | 1.3 | 199 | 191 | 194 | 218 | 1.1 |
| Vit B₃ (mg NE) | 180 | 166 | 143 | 135 | 16 | 205 | 190 | 163 | 154 | 14 |
| Vit B₅ (mg) ¥ | 23 | 23 | 23 | 30 | 5 | 23 | 23 | 23 | 30 | 5 |
| Vit B₆ (mg) | 194 | 189 | 182 | 186 | 1.7 | 220 | 214 | 206 | 211 | 1.5 |
| Vit B₉ (µg) | 148 | 149 | 152 | 179 | 400 | 148 | 149 | 152 | 179 | 400 |
| Vit B₁₂ (µg) | 152 | 180 | 121 | 13 | 2.4 | 152 | 180 | 121 | 13 | 2.4 |
| Vit C (mg) | 484 | 485 | 485 | 480 | 90 | 580 | 582 | 582 | 577 | 75 |
| Vit D (µg) | 19 | 25 | 6 | 0 | 15 | 19 | 25 | 6 | 0 | 15 |
| Vit E (mg α-TE) | 112 | 113 | 111 | 124 | 15 | 112 | 113 | 111 | 124 | 15 |
| Vit A (µg)* | 160 | 163 | 163 | 148 | 900 | 205 | 210 | 209 | 191 | 700 |
| Potassium (mg) ¥ | 155 | 154 | 154 | 168 | 3400 | 203 | 202 | 201 | 220 | 2600 |
| Calcium (mg) | 116 | 126 | 131 | 119 | 1000 | 97 | 105 | 109 | 99 | 1200 |
| Sodium (mg) ¥ | 136 | 126 | 122 | 98 | 1300 | 136 | 126 | 122 | 98 | 1300 |
| Iron (mg) | 245 | 247 | 260 | 327 | 8 | 245 | 247 | 260 | 327 | 8 |
| Iodine (µg) | 73 | 96 | 73 | 37 | 150 | 73 | 96 | 73 | 37 | 150 |
| Selenium (µg) | 192 | 185 | 152 | 139 | 55 | 192 | 185 | 152 | 139 | 55 |
| Magnesium (mg) | 130 | 127 | 132 | 162 | 420 | 170 | 167 | 174 | 213 | 320 |
| Phosphorus (mg) | 243 | 250 | 249 | 229 | 700 | 243 | 250 | 249 | 229 | 700 |
| Zinc (mg) | 105 | 99 | 101 | 100 | 11 | 144 | 137 | 139 | 137 | 8 |
| LA, 18:2 n-6 (g) | 98 | 100 | 106 | 122 | 14 | 114 | 117 | 124 | 142 | 12 |
| ALA, 18:3 n-3 (g) | 46 | 51 | 58 | 75 | 1.6 | 66 | 74 | 85 | 109 | 1.1 |

RDA (Recommended Dietary Allowance) and AMDR (Acceptable Macronutrient Distribution Range) stablished by IOM.

¥ nutrients with Adequate Intake (AI) reference values.

* Retinol (preformed Vit A).

LA: linoleic acid: ALA: linolenic acid

**Supplementary Table 6.** Nutrient percentage provided from plant, animal, or daily/eggs foods-origin in omnivorous, pesco-, and ovo-lacto-vegetarian diets.

|  | **OMNIVORE** | | | **PESCO-VEGETARIAN** | | | **OVO-LACTO-VEGETARIAN** | |
| --- | --- | --- | --- | --- | --- | --- | --- | --- |
| **Nutrient** | **Plant origin** | **Animal origin (*****)** | **Eggs and dairy origin** | **Plant origin** | **Animal origin (*)** | **Eggs and dairy origin** | **Plant origin** | **Eggs and dairy origin** |
| kcal | 78.48 | 7.24 | 14.28 | 80.93 | 2.89 | 16.18 | 84.09 | 15.91 |
| Fat (%) | 71.60 | 9.94 | 18.46 | 71.57 | 3.48 | 24.95 | 75.27 | 24.73 |
| Proteins (%) | 57.15 | 20.90 | 21.95 | 63.04 | 11.72 | 25.24 | 74.90 | 25.10 |
| A vitamin (%) | 86.27 | 0.58 | 13.15 | 85.13 | 0.50 | 14.37 | 85.56 | 14.44 |
| E vitamin (%) | 92.55 | 3.41 | 4.04 | 92.40 | 3.25 | 4.34 | 95.58 | 4.42 |
| B1 vitamin (%) | 78.93 | 9.87 | 11.20 | 85.35 | 2.99 | 11.65 | 88.44 | 11.56 |
| B2 vitamin (%) | 55.17 | 6.73 | 38.10 | 57.16 | 2.64 | 40.20 | 60.27 | 39.73 |
| B3 vitamin (%) | 58.00 | 29.64 | 12.37 | 66.51 | 17.52 | 15.97 | 81.35 | 18.65 |
| B5 vitamin (%) | 89.94 | 10.06 | - | 100.00 | - | - | 100.00 | - |
| B6 vitamin (%) | 82.06 | 10.99 | 6.96 | 86.33 | 6.10 | 7.57 | 92.13 | 7.87 |
| B8 vitamin (%) | 100.00 | - | - | 100.00 | - | - | 100.00 | - |
| B9 vitamin (%) | 91.78 | 1.88 | 6.34 | 92.48 | 1.33 | 6.19 | 93.94 | 6.06 |
| Calcium (%) | 45.66 | 1.83 | 52.51 | 45.23 | 1.23 | 53.54 | 48.66 | 51.34 |
| Iron (%) | 89.35 | 6.20 | 4.45 | 93.25 | 2.19 | 4.56 | 95.67 | 4.33 |
| Potasium (%) | 81.75 | 5.36 | 12.90 | 83.60 | 3.50 | 12.91 | 87.06 | 12.94 |
| Magnesium (%) | 86.02 | 4.38 | 9.60 | 87.47 | 2.86 | 9.67 | 90.71 | 9.29 |
| Sodium (%) | 67.15 | 16.25 | 16.60 | 74.51 | 4.03 | 21.45 | 77.71 | 22.29 |
| Phosphorus (%) | 61.34 | 10.69 | 27.97 | 63.64 | 6.35 | 30.01 | 69.80 | 30.20 |
| Copper (%) | 98.69 | 1.31 | - | 100.00 | - | - | 100.00 | - |
| Iodide (%) | 45.30 | 9.26 | 45.44 | 37.65 | 24.41 | 37.94 | 50.10 | 49.90 |
| Selenium (%) | 67.95 | 20.65 | 11.40 | 68.00 | 19.98 | 12.02 | 85.39 | 14.61 |
| Zinc (%) | 66.01 | 12.99 | 21.00 | 71.99 | 2.90 | 25.11 | 75.30 | 24.70 |
| Linoleic (%) | 97.86 | 1.93 | 0.21 | 98.55 | 1.26 | 0.20 | 99.81 | 0.19 |
| Linolenic (%) | 91.66 | 5.13 | 3.22 | 93.97 | 3.24 | 2.78 | 97.59 | 2.41 |
| Araquidonic (%) | - | 100.00 | - | - | 100.00 | - | - | - |
| Estearic (%) | 66.59 | 10.62 | 22.78 | 72.41 | 3.71 | 23.88 | 75.77 | 24.23 |
| Lauric (%) | - | 1.30 | 98.70 | 0.46 | 0.24 | 99.30 | 0.91 | 99.09 |
| Miristic (%) | 1.69 | 8.96 | 89.35 | 1.89 | 7.55 | 90.57 | 2.11 | 97.89 |
| DHA (%) | 5.15 | 94.85 | - | 5.21 | 94.79 | - | 100.00 | - |
| EPA (%) | 7.14 | 92.86 | - | 7.20 | 92.80 | - | 100.00 | - |
| PUFA | 88.47 | 7.30 | 4.22 | 91.68 | 3.59 | 4.73 | 95.37 | 4.63 |
| SFA | 41.31 | 14.45 | 44.24 | 43.99 | 2.77 | 53.25 | 46.15 | 53.85 |
| MUFA | 77.80 | 7.81 | 14.39 | 79.96 | 3.10 | 16.94 | 83.00 | 17.00 |

The values were calculated using as 100% the total value of the nutrient in each diet.

* Excluding eggs and dairy.

**Supplementary Table 7.** Total environmental fingerprint by type of diet (omnivorous, pesco-, and ovo-lacto-vegetarian diet).

|  | **kg CO₂ eq/day** | **kg CFC-11 eq/ day** | **kBq U-235 eq/ day** | **kg NMVOC eq/ day** | **Disease incidence/ day** | **CTUh (non-carcinogenic)/ day** | **CTUh (carcinogenic)/ day** | **mol H⁺ eq/ day** | **kg P eq/ day** | **kg N eq/ day** | **mol N eq/ day** | **CTUe/ day** | **Pt land use/ day** | **m³ depriv./day** | **MJ/ day** | **kg Sb eq/ day** |
| --- | --- | --- | --- | --- | --- | --- | --- | --- | --- | --- | --- | --- | --- | --- | --- | --- |
| **OMNIVORE** | | | | | | | | | | | | | | | | |
| Mean | 3.80E+00 | 3.72E-07 | 6.63E-01 | 1.44E-02 | 3.33E-07 | 9.25E-08 | 2.81E-09 | 4.43E-02 | 6.51E-04 | 3.08E-02 | 1.73E-01 | 5.91E+01 | 2.26E+02 | 1.02E+01 | 4.14E+01 | 1.87E-05 |
| SD | 9.06E-01 | 8.63E-08 | 1.69E-01 | 6.13E-03 | 8.97E-08 | 2.35E-08 | 3.45E-10 | 1.28E-02 | 1.90E-04 | 2.42E-02 | 5.15E-02 | 1.72E+01 | 8.42E+01 | 2.13E+00 | 1.07E+01 | 2.80E-06 |
| **PESCO-VEGETARIAN** | | | | | | | | | | | | | | | | |
| Mean | 3.20E+00 | 3.82E-07 | 5.80E-01 | 1.69E-02 | 2.93E-07 | 8.18E-08 | 2.46E-09 | 3.81E-02 | 5.67E-04 | 2.90E-02 | 1.36E-01 | 5.18E+01 | 1.79E+02 | 9.56E+00 | 3.94E+01 | 1.72E-05 |
| SD | 9.11E-01 | 1.25E-07 | 1.24E-01 | 1.04E-02 | 1.26E-07 | 2.52E-08 | 6.46E-10 | 1.69E-02 | 2.45E-04 | 2.47E-02 | 5.13E-02 | 1.72E+01 | 6.98E+01 | 2.06E+00 | 1.18E+01 | 4.35E-06 |
| **OVO-LACTO-VEGETARIAN** | | | | | | | | | | | | | | | | |
| Mean | 2.63E+00 | 2.70E-07 | 5.57E-01 | 8.32E-03 | 2.01E-07 | 7.68E-08 | 2.11E-09 | 2.66E-02 | 5.23E-04 | 1.58E-02 | 1.05E-01 | 4.77E+01 | 1.77E+02 | 9.75E+00 | 3.21E+01 | 1.49E-05 |
| SD | 5.18E-01 | 3.40E-08 | 6.39E-02 | 2.34E-03 | 4.91E-08 | 2.74E-08 | 4.08E-10 | 6.98E-03 | 2.14E-04 | 4.71E-03 | 2.78E-02 | 1.55E+01 | 6.27E+01 | 2.03E+00 | 6.63E+00 | 2.51E-06 |
| **VEGAN** | | | | | | | | | | | | | | | | |
| Mean | 2.07E+00 | 2.58E-07 | 5.39E-01 | 7.40E-03 | 1.47E-07 | 6.92E-08 | 1.89E-09 | 1.87E-02 | 4.77E-04 | 1.36E-02 | 7.04E-02 | 4.21E+01 | 1.51E+02 | 9.49E+00 | 3.04E+01 | 1.38E-05 |
| SD | 4.63E-01 | 3.36E-08 | 6.96E-02 | 2.16E-03 | 3.66E-08 | 2.83E-08 | 3.71E-10 | 5.23E-03 | 2.06E-04 | 4.58E-03 | 1.98E-02 | 1.70E+01 | 5.84E+01 | 2.09E+00 | 6.07E+00 | 2.54E-06 |
